# Hidden impacts of ocean acidification to live and dead coral framework

**DOI:** 10.1098/rspb.2015.0990

**Published:** 2015-08-22

**Authors:** S. J. Hennige, L. C. Wicks, N. A. Kamenos, G. Perna, H. S. Findlay, J. M. Roberts

**Affiliations:** 1 Centre for Marine Biodiversity and Biotechnology, Heriot-Watt University, Edinburgh EH14 4AS, UK; 2 School of Geographical and Earth Sciences, University of Glasgow, Glasgow G12 8QQ, UK; 3 Plymouth Marine Laboratory, Plymouth PL1 3DH, UK; 4 University of North Carolina Wilmington, Wilmington, NC 28403-5928, USA; 5 Scottish Association for Marine Science, Oban, Argyll PA37 IQA, UK

**Keywords:** ocean acidification, cold-water corals, climate change, biomineralization, calcification, *Lophelia pertusa*

## Abstract

Cold-water corals, such as *Lophelia pertusa*, are key habitat-forming organisms found throughout the world's oceans to 3000 m deep. The complex three-dimensional framework made by these vulnerable marine ecosystems support high biodiversity and commercially important species. Given their importance, a key question is how both the living and the dead framework will fare under projected climate change. Here, we demonstrate that over 12 months *L. pertusa* can physiologically acclimate to increased CO_2_, showing sustained net calcification. However, their new skeletal structure changes and exhibits decreased crystallographic and molecular-scale bonding organization. Although physiological acclimatization was evident, we also demonstrate that there is a negative correlation between increasing CO_2_ levels and breaking strength of exposed framework (approx. 20–30% weaker after 12 months), meaning the exposed bases of reefs will be less effective ‘load-bearers’, and will become more susceptible to bioerosion and mechanical damage by 2100.

## 1. Introduction

Cold-water corals (CWCs), such as *Lophelia pertusa,* form complex three-dimensional framework that support high biodiversity [1,2] and commercially important species [3]. These vulnerable marine ecosystems [4] are found throughout the world's oceans to 3000 m deep [1,2]. Projections of ocean acidification suggest that ocean pH will decrease by another approximately 0.3–0.4 pH units by the end of the century [5] and decrease the saturation state of aragonite (calcium carbonate polymorph). The cold-water Scleractinia of the deep-sea live at lower temperature (4–12°C) [2] and aragonite saturation states (*Ω*_Aragonite_) than tropical species. It is estimated that up to 70% of CWC reefs, which currently live at low saturation states relative to tropical corals (*Ω*_Aragonite_ < 2) will be in aragonite-undersaturated water by the end of the century, and as such are at greater risk than tropical Scleractinia from the projected shallowing of the aragonite saturation horizon [2,5,6]. Given their importance, [4] a key question is how both the living and the dead framework will fare under projected climate change [7].

**Figure 1.**
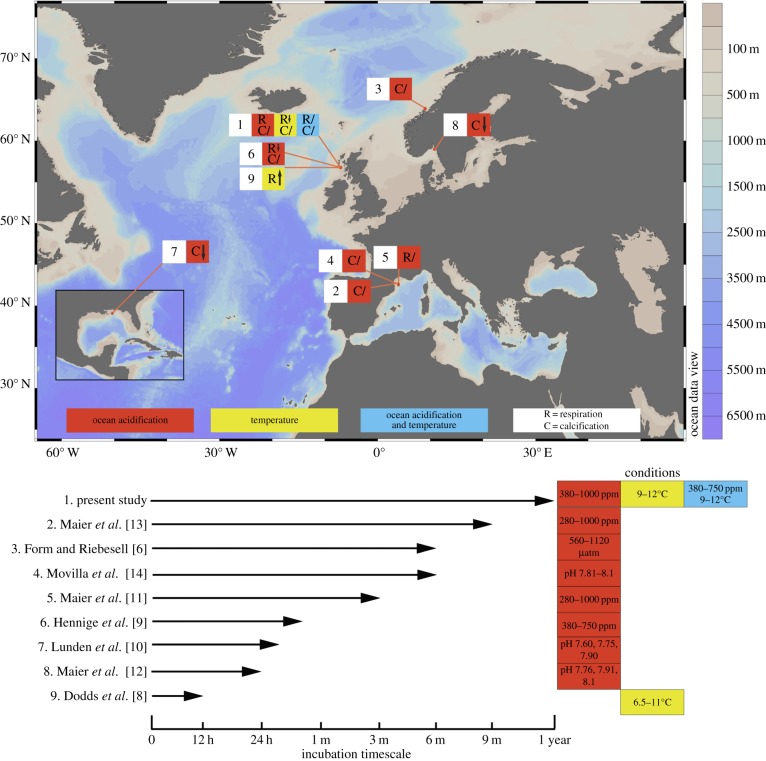
Chart showing locations and summarizing physiological results from research on projected future impacts of temperature and ocean acidification on *Lophelia pertusa*. ‘R’, Respiration; ‘C’, calcification; ‘↑’, an increase; ‘↓’, a decrease; ‘/’, no statistically significant change. Symbols represent experiment endpoint results, pH is recorded in the total scale.

Studies have examined the response of the abundant CWC *L. pertusa* to single stressors, ocean acidification or warming, using coral respiration or calcification rates as response variables [6,8–14]. The general consensus was that considerable variability exists between individuals [10], but *L. pertusa* has the ability to tolerate single stressors (figure 1). However, there are major knowledge gaps to be explored before any inferences can be made as to the long-term survival and ecological role of CWC reefs [15]. Since the most likely future climate scenario involves changes in both temperature and CO_2_ [16], it is vital to understand whether CWCs can acclimatize to multiple stressors simultaneously, and whether this is at a cost to other processes [17]. While adaptation to changing conditions can occur over subsequent generations, the slow growth of CWCs coupled with the projected rapid change in ocean acidification and warming [18], means that reef survival will depend heavily on the acclimatization capacity of currently living CWCs. Their acclimatization capacity is their ability to respond plastically to their environment [19] and is genetically constrained [20]. To assess acclimatization ability, it is vital to conduct relatively long-term experiments, as short-term experiments may produce results (e.g. detrimental impacts of ocean acidification upon key processes), which may not appear in long-term studies, as organisms have undergone alterations in key regulatory processes to acclimatize [19]. While *L. pertusa* carbon budgets are not fully understood due to limitations of obtaining and experimenting on live samples, energetic inputs (food) and reserves will be used for calcification, respiration, reproduction, maintenance, and particulate and dissolved organic matter release (electronic supplementary material, figure S1).

Even if corals can acclimatize sufficiently, net reef accretion in aragonite-undersaturated conditions (*Ω*_Aragonite_ < 1) will only occur if coral calcification exceeds dissolution and bioerosion of exposed dead skeleton, and if skeletons can continue to physically support the living reefs sitting above them. This is critical to understand, since the coral's skeletal framework provides an important ecosystem function, and may persist for millennia. To explore these issues, we conducted a 1-year study on *L. pertusa* to address three main questions in a long-term experimental context:

(1) Can *L. pertusa* acclimatize to elevated temperature and CO_2_ with continued calcification and respiration at present-day rates?
(2) Do elevated temperature and CO_2_ conditions impact the biomineralization of newly produced skeleton?
(3) Does ocean acidification have a detrimental impact on the strength of exposed *L. pertusa* skeletons?

## 2. Material and methods

### (a) Sample collection

Medium to large colonies of *L. pertusa* 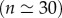 were collected from Area 1 within the Mingulay Reef Complex [1,9,21], 56°49.38 N, 7°22.56 W during the RRS *Discovery* cruise 366/7 in July 2011 [22]. Colonies were collected using a modified video assisted van-Veen grab [23] from 141 to 167 m.

Corals were placed in holding tanks at ambient seabed temperature. Upon return to laboratory, corals were gradually acclimatized to inshore seawater (temperature controlled at 9°C) collected from the east coast of Scotland. Following two months acclimatization, corals were then carefully fragmented into smaller fragments with 5–20 polyps, taken from the top of sampled colonies to ensure that relatively young polyps were used. Fragments were attached to PVC pipe bases with Grotech Korafix epoxy, and split between experimental systems (to prevent treatment/colony pseudoreplication) kept at ambient conditions for two months prior to experimentation.

Experimental conditions replicated collection site ambient and predicted future conditions following IPCC IS92a emission scenarios. Experimental tank temperatures and the CO_2_ mixes bubbled into the tanks were: (i) 9°C 380 ppm (the ambient environment); (ii) 9°C 750 ppm (ambient temperature, elevated CO_2_); (iii) 12°C 750 ppm (elevated temperature, elevated CO_2_); (iv) 12°C 380 ppm (elevated temperature and ambient CO_2_); and (v) 9°C 1000 ppm (ambient temperature, elevated CO_2_). All treatments are referred to in results and discussion by the target temperatures and the input CO_2_ for ease of reference, rather than their calculated *p*CO_2_ (see the electronic supplementary material, table S1).

For each treatment, there were four replicate systems, each comprising four 5 l tanks connected to a 60 l sump. Each tank was suitable for holding *n* = 4 live coral fragments and a ‘dead’ coral skeleton (80 tanks total). Each replicate system held approximately 80 l seawater. Ambient and elevated CO_2_ air mixes were bubbled directly into the sump. Gas mixing was achieved in-house, and analysed continuously with a gas analyser (Licor-820) calibrated using pre-mixed 0 and 750 ppm CO_2_ gases (StG gases). Target experimental values were checked and corrected daily. All replicate systems were housed within a temperature-controlled room at ambient reef temperature (9°C ± 0.5°C), and systems at elevated temperatures were controlled through Aqua Medic T-computers and titanium heaters at 12°C ± 0.5°C (Aqua Medic TH-100). Bubbled sumps were also equipped with filtration units and powerheads to ensure adequate filtration and water mixing for each replicate system. To check water parameters and ensure target pH levels were reached from bubbling, tank pH, salinity and temperatures were measured with a Mettler-Toledo SevenGo SG2 pH meter and a YSI (30) salinity and temperature meter. Average pH_(NBS)_ values for each treatment over the experiment duration were 9°C 380 ppm = 8.01 (s.d. ± 0.02); 12°C 380 ppm = 8.00 (s.d. ± 0.03); 9°C 750 ppm = 7.89 (s.d. ± 0.02); 12°C 750 ppm = 7.91 (s.d. ± 0.04) and 9°C 1000 ppm = 7.78 (s.d. ± 0.05). Corals were fed a mixture of live *Artemia* and crushed krill (Gamma) every 2 days, and 20% water changes were conducted on each replicate system once per week. Following measurements at time zero, temperatures and CO_2_ levels were increased to target levels over two weeks. Experimental time points were time zero, +3 months, +6 months and +12 months.

### (b) Metabolism and calcification rates

Rates of oxygen consumption (µmol O_2_ g^−1^ ash-free dry mass (AFDM) h^−1^) and calcification rates (µmol CaCO_3_ g^−1^ h^−1^) were assessed for coral fragments placed within 200 ml incubation chambers fitted with oxygen optodes connected to a temperature-compensated oxygen analyser (Oxy-4 Mini with Temp-4, Presens & Loligo systems) [9]. Sensor spots were each calibrated against air-saturated water and oxygen-free water. Magnetic stirrers ensured homogeneity of oxygen around the coral fragments in each incubation chamber. Chambers were arranged around a central magnetic stir controller to ensure that the stir bars within chambers, and hence the flow rates, were consistent between all chambers. All chambers were filled with tank-specific seawater and corals were allowed to acclimate to the conditions for 2 h prior to measurements. Ten chambers were used per treatment at each time point; eight for respiration and calcification measurements on live coral fragments and two as seawater ‘blanks’ in order to measure (and subsequently subtract) background rates. Prior to measurements, corals were not fed for 48 h. Following respiration and calcification rate measurements, the AFDM of each sample was determined by adding homogenized material to a pre-weighed porcelain crucible and placed in a muffle furnace at 450°C for 4 h. Crucibles were re-weighed and the difference in the weight gave the amount of organic matter or AFDM. Calcification rates were calculated following alkalinity anomaly techniques from Smith & Key [24] and Ohde & Hossain [25], by measuring the change in seawater alkalinity after 4 h in the respiration chambers using the equation calcification = 0.5 (ΔTA)·*V*/Δ*T*/AFDM, where ΔTA is the change of total alkalinity (mmol/l), *V* is the volume of experimental seawater (*L*) and Δ*T* is the experimental period (h).

Nutrients changes were considered negligible in this study, as the approximately 10% underestimation of net calcification rate due to nutrient omission is small compared with natural variation in CWC calcification [26]. Changes in aragonite saturation state, dissolved inorganic carbon (C_T_) and pH during incubation were not quantified during incubations. For respiration and alkalinity anomaly measurements, it is likely that there will have been a significant rise in C_T_ and corresponding decrease in pH in the incubation chambers during the respiration incubation periods [6,11]. Although unquantified, reductions in pH may have been similar to those reported by Maier *et al.* [11,12]. However, there was no difference in respiration rates between 380 and 1000 ppm ambient temperature treatments, and respiration rates do not differ in incubation chambers over a 4-h time period (SJ Hennige 2012, personal observation). Therefore, potential reductions in pH during incubations are likely to have negligible impact compared to long-term treatment acclimatization.

### (c) Corallite shape

To assess whether newly grown corallites differed between treatments, corallites grown within the experimental time frame (that were not present at the experiment start, as identified by comparing pictures from the experiment start and the experiment end) were embedded in EpoHeat Epoxy (Buehler). Blocks of resin containing the coral sample were then sliced until the middle of the new corallite was sectioned. Photographs were taken and Image J [27] was used to determine corallite height (from middle tip of new corallite growth to the middle base of the new corallite), and width at the base of the new corallite.

### (d) Biomineralization

To image the surface of whole skeletal fragments, and also for electron back scatter diffraction (EBSD), an FEI Quanta 200F field emission scanning electron microscope (SEM) equipped with a TSL EBSD system was used [28]. To assess the molecular-scale bonding of the skeleton, a Renishaw inVia Raman spectrometer equipped with a Leica DM 2500 M microscope and 785 nm laser, with the aragonite peak centred at 1085 cm^−1^ was used [28]. Full-width half-maximum (FWHM) measurements of the aragonite peak at 1085 cm^−1^ were collected at the outside tips of newly grown corallites, as close as possible to the sample edge (approx. 5 µm).

### (e) Ecologically relevant breaking strength

At the end of the experimental period, ‘dead’ coral skeleton fragments (tissue removed at experiment start) were collected from each replicate treatment, and matched according to size to reduce variability (*n* = 5 per treatment). Branches were cut cross-sectionally using cutting discs in a Dremel, and bases were embedded in EpoxiCure 2 Resin (Buehler) within a 25 mm copper tube to prevent resin flex during testing. The mean coral length extending from the resin was 7.57 mm ± s.e. 0.56. Diameters of all samples were recorded, and wall thicknesses were assessed following breaking using a binocular microscope.

Ecologically relevant breaking strength was tested using a Zwick Roell Z2.0 materials test instrument. Samples were clamped in a vice and exerting load applied perpendicularly to the sample at 2 mm min^−1^ at a consistent distance from the base for each sample. Force was applied until the samples broke, and TestXpert II software was used to analyse results. Applied force (N) was normalized to individual coral diameter (D) (average of 7.32 mm ± s.e. 0.69) as N/D, and to wall thickness (Wt) (average of 0.88 mm ± s.e. 0.14) combined with diameter as N/(D/Wt), to reduce the effect of natural variability in coral sizes between fragments.

### (f) Carbonate chemistry of experimental systems

At experimental time points, seawater was collected in borosilicate glass bottles with ground glass stoppers (100 ml) from replicated systems for each treatment. Sample bottles were rinsed, filled and poisoned according to standard procedures detailed in Dickson *et al*. [29].

Total dissolved inorganic carbon (C_T_) was measured using an automated Apollo SciTech Dissolved Inorganic Carbon Analyser. Total alkalinity (A_T_) was measured on an Apollo SciTech Alkalinity Titrator Model AS-ALK2 using the open-cell potentiometric titration method. Calibration was made using Certified Reference Materials with a repeatability of max ± 0.1% for C_T_ and A_T_. For additional measurements (and for the alkalinity anomaly technique), A_T_ was measured using an automatic titrator (Metrohm 702 SM Titrino). The open-cell potentiometric titration method was used with 20 ml sample volumes and 0.01 M HCl. All A_T_ samples were analysed at 25°C (±0.1°C) with temperature regulation using a water-bath (Grant OLS 200). Certified Reference Materials (batch 109) from A. G. Dickson (Scripps Institution of Oceanography) were used to standardize at the beginning and end of each day of analysis, with repeatability of max ± 0.13%. Carbonate parameters were calculated using CO2calc with dissociation constants [30], refit by Dickson & Millero [31] and KSO_4_ using Dickson [32].

### (g) Statistics

For data that met normal distribution assumptions (Kolmogorov–Smirnov) and equal variances (Levene), analyses of variance (ANOVA) were used with post hoc tests (Tukey). Where normality assumptions were not met, Kruskal–Wallis tests were used. To assess potential correlations between coral breaking strength and experimental *p*CO_2_ conditions, a Pearsons two-tailed correlation was used once normality had been tested and met. Extremes in corallite shapes were compared using a two-tailed *t*-test. All statistical analysis was conducted in Prism v. 5.0c. for Mac, GraphPad Software, San Diego, CA, USA.

## 3. Results

**Figure 2.**
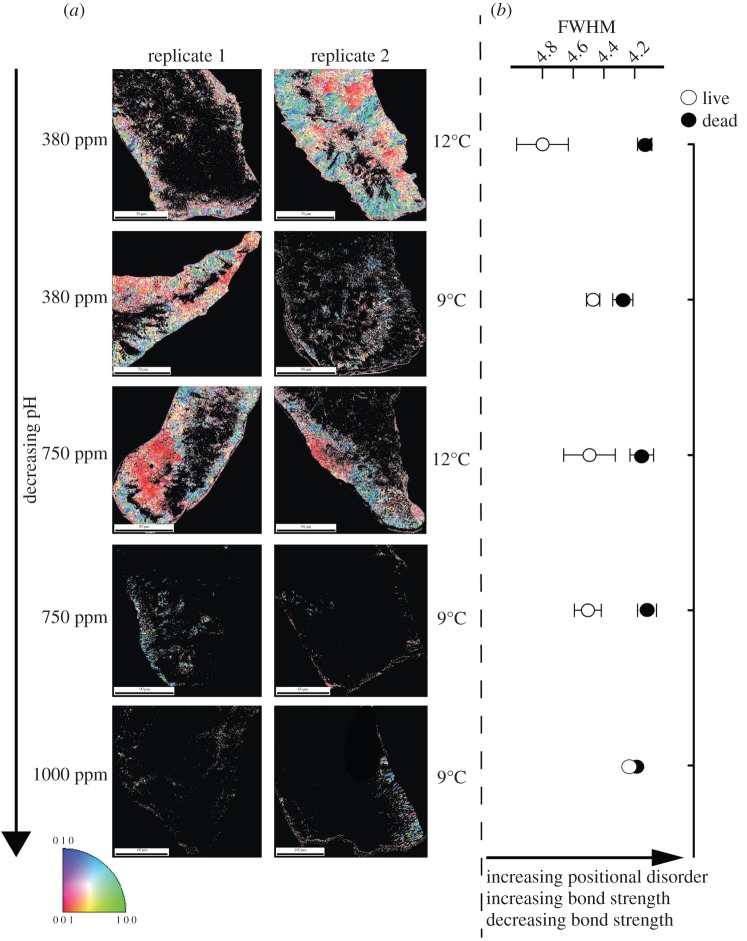
(*a*) EBSD of *Lophelia pertusa* calcification during the year-long experiment. Colours indicate grouped crystal organization and orientation. (*b*) Full-width half-maximum (FWHM) of aragonite peak spectra at *ca* 1085 cm^−1^ of skeleton from living, or unprotected dead *L. pertusa*.

Over 12 months, there were significant effects of increased temperature and CO_2_ upon new corallite biomineralization, shape and molecular-scale bonding organization. Microstructural analysis of the skeleton demonstrated that under ambient CO_2_, corals have well-organized aragonitic ‘bundles’ as indicated by high diffraction and identifiable crystal orientations throughout the majority of the sample (figure 2*a*; electronic supplementary material, figure S2) [33]. Darker areas are also visible within the sample and represent centres of calcification (COC)/early mineralization zones [33,34]. Under high CO_2_ conditions (750 or 1000 ppm CO_2_; figure 2*a*), diffraction decreases throughout the samples, so dark areas are more prominent.

**Figure 3.**
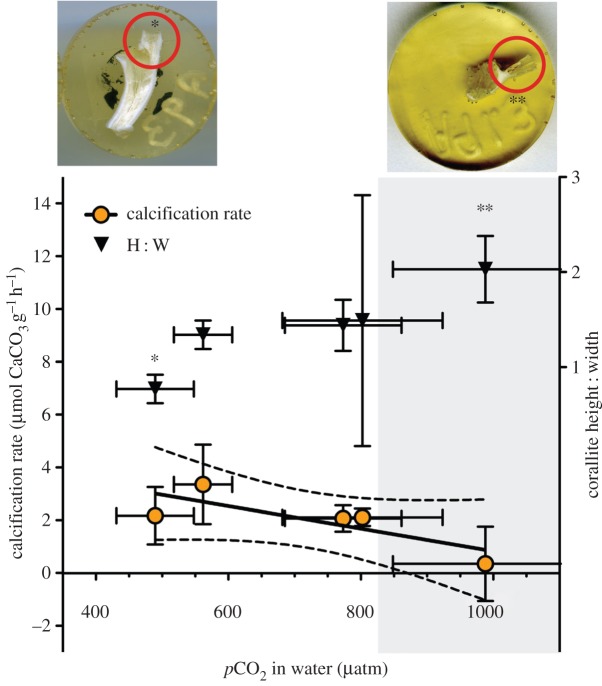
Calcification rates ±s.e. of *Lophelia pertusa* expressed as µmol CaCO_3_ g^−1^ dry tissue h^−1^ versus adjusted *p*CO_2_ with linear trend ± 95% CIs at *T* + 12 months. Black triangles represent aspect ratio ±s.e., of newly formed corallites, with two example images of control and high CO_2_ treatments that significantly differed. The shaded area represents water conditions *Ω*_Aragonite_ < 1. Asterisks (* and **) denote example corallites represented by the data.

**Figure 4.**
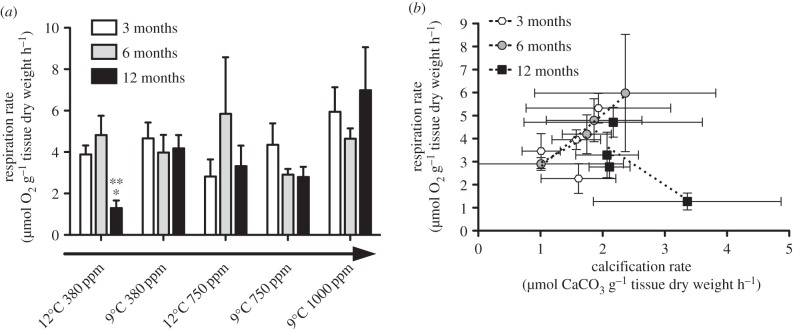
(*a*) Respiration rates ±s.e. of *Lophelia pertusa* expressed as µmol O_2_ g^−1^ AFDM h^−1^ for each treatment at three, six and 12 month time points. Asterisks (* and **) denote significant differences at that time point between and within treatments, respectively. The arrow indicates a decrease in pH across treatments. (*b*) Respiration and calcification rates ±s.e. for all treatments at three, six and 12-month time points. Linear regression lines are fitted for each time point between treatments. Nine degrees Celsius 1000 ppm treatment results are excluded due to dissolution of exposed aragonite.

The same trend was not evident in fragments from elevated temperature *and* elevated CO_2_ treatments, where good diffraction was observed (figure 2*a*). Crystal molecular bond length [35,36] also decreased under high CO_2_ conditions (figure 2*b*), with significantly higher positional disorder (lower FWHM) of the aragonite in 9°C, 1000 ppm corallites compared to 12°C 380 ppm corallites at 12 months (one-way ANOVA with Tukey; *F*_4,10_ = 3.257, *p* = 0.059, with post hoc showing significant change between 9°C, 1000 ppm and 12°C 380 ppm, *Q* = 5.09. *p* < 0.05). FWHM was also significantly lower in exposed skeletons than in tissue-protected skeletons (figure 2*b*) (*F* = 22.3, d.f. = 20, *p* < 0.001). New corallites grown at 1000 ppm were significantly longer and thinner than under ambient conditions (*t* = 3.31, d.f. = 5, *p* = 0.02; figure 3). Absolute calcification rates of corals under all treatments did not change significantly (figure 3 and table 1), although dissolution of exposed areas of skeleton on corals is evident where *Ω*_Aragonite_ < 1, with average calcification data near zero (figure 3) due to some individuals having net dissolution. Respiration rates (within ranges described by Dodds *et al.* [8] and Maier *et al.* [11], and lower than those of Hennige *et al.* [9], where respiration rates were measured at sea on freshly collected *L. pertusa*) did not significantly differ in corals under ambient or high CO_2_ conditions over three, six or 12 months (figure 4*a*; electronic supplementary material, figure S3). The only significant change in respiration rates were observed in corals exposed to the single stressor of elevated temperature after 12 months, with a significant reduction in respiration both within and between treatments (within treatment, Kruskal–Wallis with Dunn's multiple comparison test, *H* = 9.89, *p* = 0.04; between treatments, *H* = 8.05, *p* = 0.02; figure 4*a*; electronic supplementary material, figure S3). Respiration was also plotted against calcification (figure 4*b*); there was a significant positive linear regression (*r*^2^ = 0.610, *p* = 0.02) when three and six month data were combined. The regression was non-significant (*r*^2^ = 0.02, *p* = 0.66) with the inclusion of 12 month data. Nine degrees Celsius, 1000 ppm data points were not included due to dissolution of exposed aragonite during calcification measurements.

**Table 1.** Calcification rates of *Lophelia pertusa* (±s.e., *n* = 8) measured at different experimental time points as µmol CaCO_3_ g^−1^ dry tissue mass h^−1^.

	calcification rate (µmol CaCO_3_ g^−1^ dry tissue h^−1^)				
time (month)	9°C, 380 ppm	12°C, 380 ppm	9°C, 750 ppm	12°C, 750 ppm	9°C, 1000 ppm
3	1.93 (1.16)	1.58 (0.39)	1.01 (0.31)	1.61 (0.60)	0.22 (0.64)
6	1.74 (0.39)	1.21 (0.32)	1.01 (1.33)	2.36 (1.46)	0.21 (1.54)
12	2.17 (1.09)	3.36 (1.51)	2.11 (0.33)	2.07 (0.50)	0.35 (1.41)

**Figure 5.**
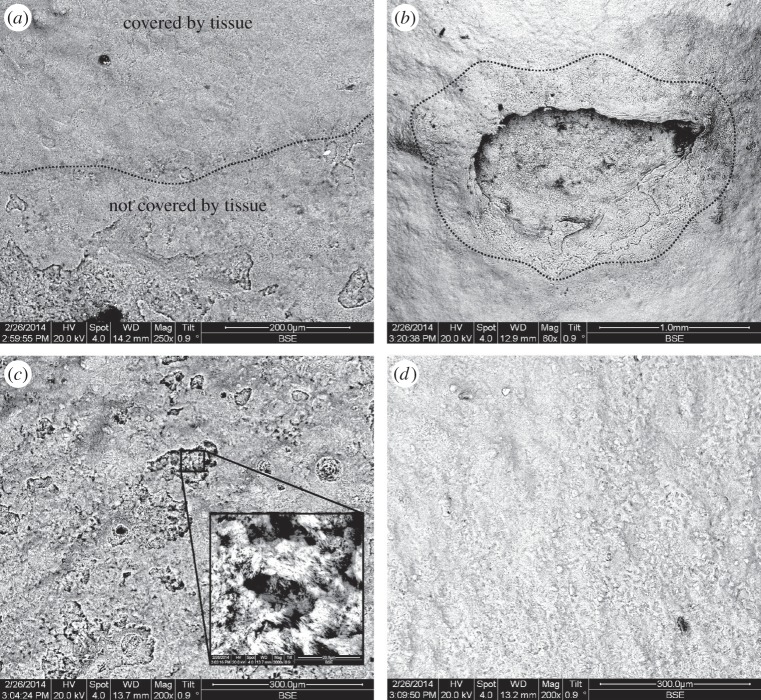
Back scattered electron emission of *Lophelia pertusa* skeleton fragments maintained in *Ω*_Aragonite_ < 1. (*a*) The interface (dashed line) between tissue-protected skeleton (top) and exposed skeleton (bottom). (*b*) A site of tissue damage on *L. pertusa*, and subsequent dissolution of skeleton in an otherwise protected area. (*c,d*) Exposed and tissue-protected sections of skeleton, respectively, with close-up inset.

Exposed coral skeletons from high CO_2_ treatments where *Ω*_Aragonite_ < 1 exhibited substantial dissolution and ‘pitting’ when viewed under SEMs (figure 5). This was evident in areas at the base of corals where tissue was retracted, and in areas of tissue damage (figure 5). A significant negative correlation was found between breaking strength of exposed skeletons and CO_2_ (table 2), such that exposed coral framework was approximately 20–30% weaker after 12 months exposure (Pearson's correlation for *N* normalized as *N*/*D* = −0.82, *p* = 0.04, *r*^2^ = 0.68; Pearson's correlation for *N* normalized as *N*/(*D*/Wt) = −0.97, *p* = 0.01, *r*^2^ = 0.95).

**Table 2.** Force (*N*) required to break coral skeletons (*n* = 5 ± s.e.) exposed to treatments for 12 months with treatment *p*CO_2_ (µatm) and saturation of aragonite (*Ω*_Aragonite_) normalized to coral diameter (*D*), and coral diameter and wall thickness (*D*/Wt).

treatment	*p*CO_2_ µatm	*Ω*_Aragonite_	breaking force (*N*/*D*)	breaking force (*N*/(*D*/Wt))
9°C 380 ppm	489.4 (58.4)	1.56 (0.15)	28.0 (3.66)	25.9 (3.66)
12°C 380 ppm	562.0 (44.1)	1.49 (0.20)	25.0 (3.79)	22.1 (1.20)
9°C 750 ppm	802.5 (121.0)	1.09 (0.16)	17.8 (4.25)	17.2 (5.28)
12°C 750 ppm	773.4 (88.1)	1.19 (0.13)	22.5 (7.54)	20.0 (6.94)
9°C 1000 ppm	988.0 (139.6)	0.76 (0.10)	20.5 (1.49)	13.1 (3.78)

## 4. Discussion

### (a) Long-term acclimatization and changes in biomineralization

Under ocean acidification scenarios (single or combined stressors), there was no significant difference in calcification or respiration rates from controls at three, six or 12 months. Both of these processes usually positively correlate [11], and this was observed here at three and six months. However, this relationship breaks down with the inclusion of 12-month data. Considering that these energetically costly processes are usually coupled, a deviation from this relationship may indicate that ‘normal’ energetic strategies are no longer applying, possibly due to other processes using energetic reserves. The significant reduction in respiration at 12 months in corals in the elevated temperature treatment may represent such a change, and highlights that many processes may be occurring of which we have poor understanding and/or cannot easily measure.

In the most likely future climate scenario, where both temperature and CO_2_ levels increase, calcification and metabolic rates of *L. pertusa* do not differ from controls. In all climate change studies to date on *L. pertusa*, significant changes in respiration and calcification are only seen in the short term (figure 1), from 24-h experiments to four weeks. Beyond four weeks, changes are not observed (figure 1) [6,11,13,14]. While this study did not observe any decrease in calcification or respiration at the earliest time point (three months), comparable shorter-term studies performed on *L. pertusa* collected on the same expedition did observe significant impacts of ocean acidification on respiration [9]. Given that *L. pertusa* has been demonstrated to be significantly impacted by ocean acidification in short-term exposures [6,9,10,12], we can infer from non-significant differences here between controls and ocean acidification treatments that corals have acclimatized to their new conditions. It is likely that in the days to months following a perturbation, energetic pathways in this species may prioritize protective or acclimation pathways (e.g. induction of heat shock proteins [17]). It is currently unknown whether energetic costs are involved in changing corallite shape and crystal molecular bonding, or whether this is a consequence of reducing resource allocation to cytoskeletal organization (which may vary by species [37]), but this question remains a priority for future investigations.

Here, it was demonstrated that crystal organization changes under elevated CO_2_ levels. Under control conditions and under elevated temperatures, typical diffraction and organization of skeletal aragonite was observed. As CO_2_ increased, diffraction decreased and only scattered and disjointed aragonite crystals were imaged. The COC typically acts as the biomineralization ‘scaffold’ [34] and is surrounded by fibrous aragonitic bundles. The decrease in diffraction observed here indicates that a change has occurred to biomineralization processes under high CO_2_ leading to less organization in the fibrous aragonite crystals. Interestingly, corals that were exposed to elevated temperature and CO_2_ did not show the same crystal disorganization as corals exposed to high CO_2_ alone. Evidence is now emerging in tropical corals that organic matrix protein incorporation in coral skeletons, which may promote calcification in less favourable calcifying fluid conditions [38], significantly changes under elevated CO_2_ conditions. Along with results here, it highlights the need for multi-parameter analysis of calcification and biomineralization in an ocean acidification and warming ocean context. Multi-stressor experiments are also very important, as increased temperature in conjunction with ocean acidification may impact biomineralization differently than ocean acidification alone, such as demonstrated in crystal organization here.

It is noteworthy that *L. pertusa* is potentially a relatively plastic coral species as demonstrated by its large bathymetric range spanning a variety of aragonite saturation states (30–3000 m [1,2]), its occurrence in different temperature habitats [6,26,28], and its ability to thrive in areas such as the Mingulay Reef Complex, where tidally driven downwellings cause daily variations in carbonate chemistry equivalent to *ca* 25 year jump in atmospheric CO_2_ [39]. The growth of longer and thinner corallites in high CO_2_ treatments might represent one plastic ability of *L. pertusa* to alter morphology. Longer and thinner corallites could enhance prey capture opportunities for individual polyps [40], which would be key to meeting potentially increased acclimation costs under high CO_2_ conditions. However, such corallites may be more easily damaged or snapped, thereby decreasing future framework stability.

### (b) The future of deep-sea coral reefs

It is encouraging that the CWC *L. pertusa* appears to be able to physiologically acclimate to future projected environmental changes. However, this by no means guarantees their long-term survival. Acclimatization comes at a cost, and this cost either has to be met by increased energetic inputs [41], or by re-allocation of energy from other processes. Some of these costs may be met by changes to biomineralization processes and needs to be investigated further, but the change observed in the linear relationship between respiration and calcification indicates that changes could be happening in energetic allocation. The reduction in respiration shown by corals exposed to high-temperature treatment after 1 year also indicates that processes may be changing, and highlights that many processes may be occurring of which we currently have poor understanding. Of great concern is that all studies to date on *L. pertusa* do not account for any impact on coral reproductive fitness. Indeed, Albright & Mason [42] demonstrated how the fertilization success of a tropical coral was negatively impacted by increased CO_2_. Acclimatization may therefore not ensure the long-term survival of corals if reproductive fitness also decreases.

Another great concern for the future of deep-water coral reefs is the dissolution of exposed skeleton if the surrounding water becomes undersaturated with respect to aragonite, with a corresponding reduction in load-bearing capacity. While extracellular pH upregulation of the calcifying fluid allows corals to actively grow in undersaturated water [43], this does not protect exposed skeleton [44], which frequently forms the largest proportion of any deep-water reef framework [1]. Although the imperforate skeletons of *L. pertusa* will likely have slower dissolution rates than many perforate corals [44], and continuous framework may be colonized and potentially ‘protected’, bio-eroding sponges may become more efficient under future conditions thereby weakening framework colonized by other epifauna further [45]. While the ecologically significant ability of adult *L. pertusa* to skeletally fuse [28] helps strengthen the framework as a whole, the fact that over 95% of CWC reefs are found above the saturation horizon depth [5] infers that, in the long-term, net reef growth cannot normally be maintained in undersaturated water. The importance of skeletal dissolution with regard to ocean acidification has largely been overlooked in the discussion on how coral ecosystems will fare under future climate change [46]. Importantly, given that no adaptation can happen with regard to dissolution as it is a biogeochemical response [46], it is potentially increased dissolution of exposed aragonite, rather than a reduction in calcification rates of the live coral that could lead to future net CWC reef loss. A loss of positive reef accretion has been observed in tropical coral ecosystems along natural CO_2_ gradients [47], and it is feasible to assume that a similar reduction would happen in CWC reefs as the aragonite saturation horizon shoals.

Overall, we demonstrate here for the first time that *L. pertusa* can acclimatize to multiple stressors of temperature and CO_2_, but that significant changes happen to its skeletal biomineralization, molecular-scale bonding and structure. We also demonstrate that exposed coral framework, which forms the structural base of CWC reefs becomes structurally weaker even after 12 months of high CO_2_ conditions. The question remains as to whether *L. pertusa* can continue to calcify at a rate which supports net reef growth, or whether potential energetic reallocation from key processes will result in decreased fitness and a long-term loss in ecosystem function when combined with weakening and dissolution of their foundation framework. Given our new evidence that CWC biomineralization changes under projected conditions, and that exposed framework becomes weakened, it is premature to assume that the impacts of ocean acidification on CWCs will be minimal based solely on the ability of the live coral to physiologically acclimatize in the short term. Strategies to reduce CO_2_ emissions are still needed to minimize impacts of ocean acidification on CWCs as well as other marine biodiversity [18].

## Supplementary Material

Hidden impacts of ocean acidification to live and dead coral framework: Supplementary Information

## Data accessibility

All data are deposited at the British Oceanographic Data Centre [48].
