# Supplementary material for: Hidden impacts of ocean acidification to live and dead coral framework
Source: Proc Biol Sci. 2015 Aug 22;282(1813):20150990. doi: 10.1098/rspb.2015.0990 (PMC4632617; doi:10.1098/rspb.2015.0990)
Supplement: Hidden impacts of ocean acidification to live and dead coral framework: Supplementary Information [file rspb20150990supp1.pdf]

Hidden impacts of ocean acidification to live and dead coral framework:

Supplementary Information

Hennige S.J.[1*], Wicks L.C.[1], Kamenos N.A.[2], Perna G.[2], Findlay H.S.[3], Roberts J.M.[1,4,5]

[1]Centre for Marine Biodiversity and Biotechnology, Heriot-Watt University, Edinburgh, EH14 4AS, UK

[2]School of Geographical and Earth Sciences, University of Glasgow, Glasgow, G12 8QQ, UK

[3]Plymouth Marine Laboratory, Prospect Place, West Hoe, Plymouth, England PL13DH, UK

[4]Center for Marine Science, University of North Carolina Wilmington, 601 S. College Road, Wilmington, NC 28403-5928, USA

[5]Scottish Association for Marine Science, Scottish Marine Institute, Oban, Argyll, PA37-IQA, UK

Keywords: Ocean Acidification; Cold-Water Corals; Climate Change; Biomineralisation; Calcification; *Lophelia pertusa*

*Corresponding author: S.Hennige@hw.ac.uk

Tel: +44 (0) 131 451 3148

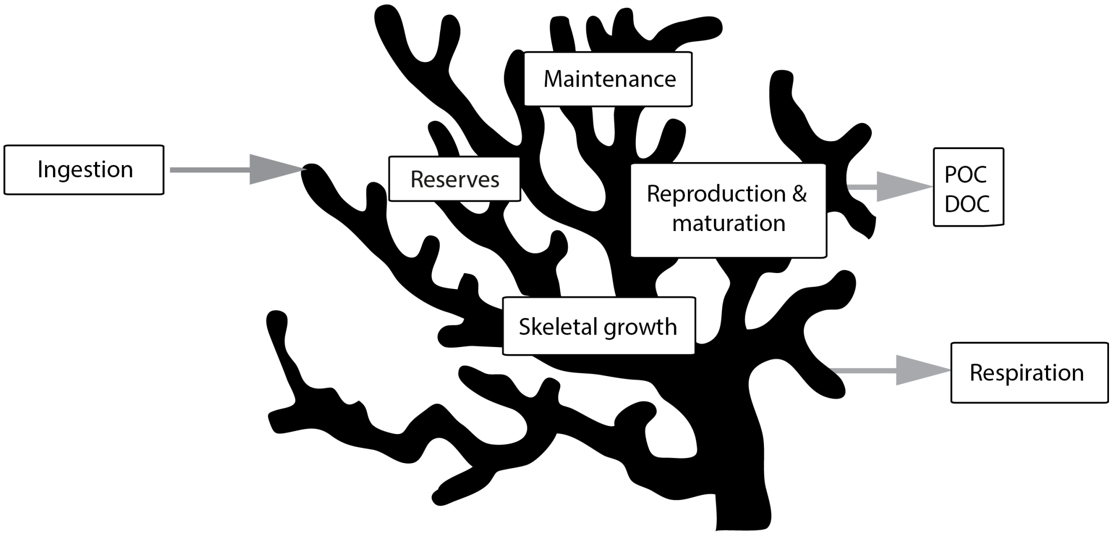

Supplementary Information Figure 1: *Simplified schematic of carbon energetic budget in Lophelia pertusa. Energetic inputs are through injestion of food sources to be used in downstream processess and in building up energetic reserves. POC and DOC stand for particulate and dissolved organic matter respectively.*

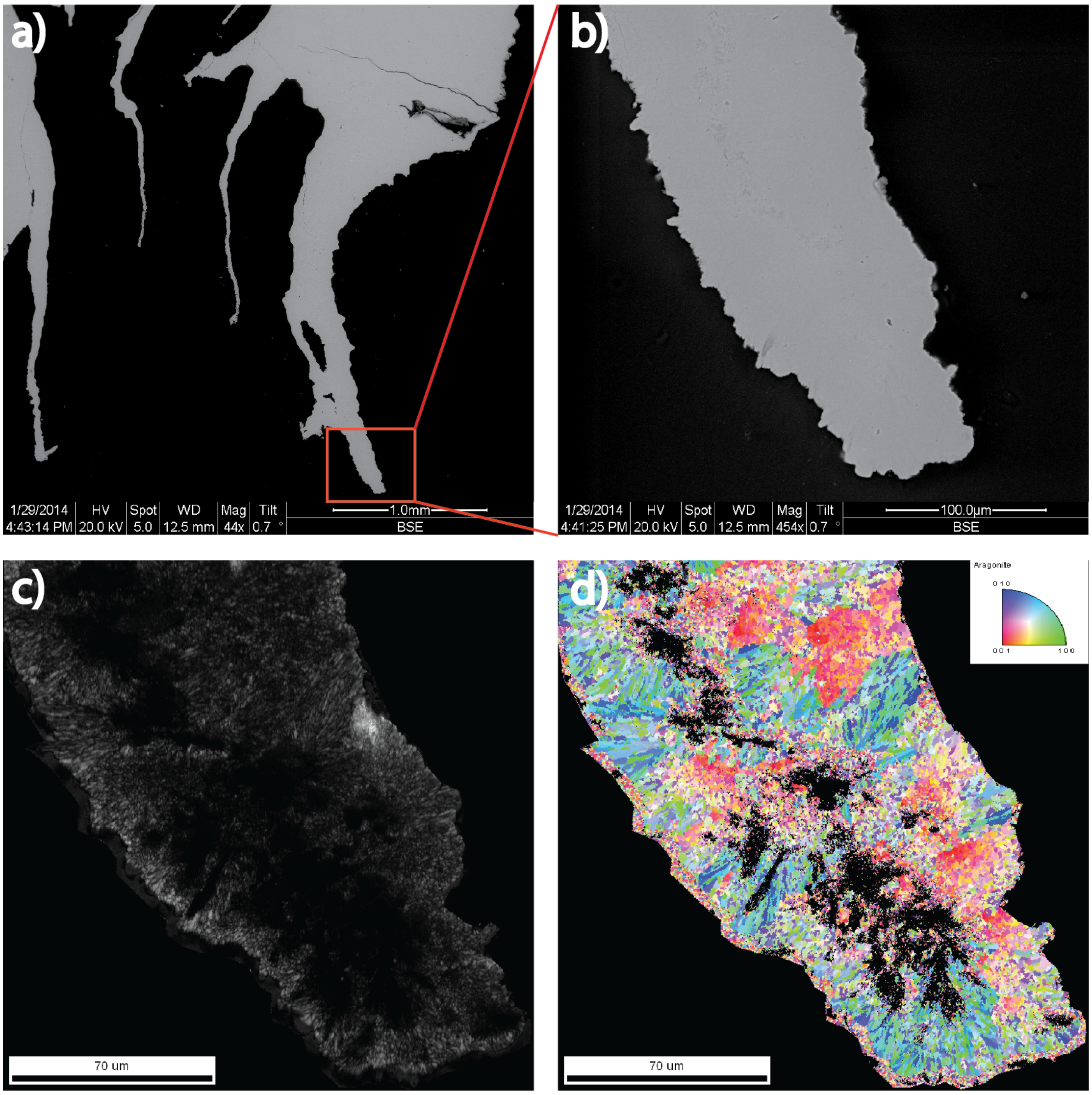

Supplementary Information Figure 2: ***(a)*** *Back Scatter Electron emission (BSE) image of polyp (grey), and* ***(b)*** *magnified area of interest of polyp tip.* ***(c)*** *Crystal Diffraction map and* ***(d)*** *orientation of aragonite crystals respectively. Colours in* ***(d)*** *indicate crystal orientation.*

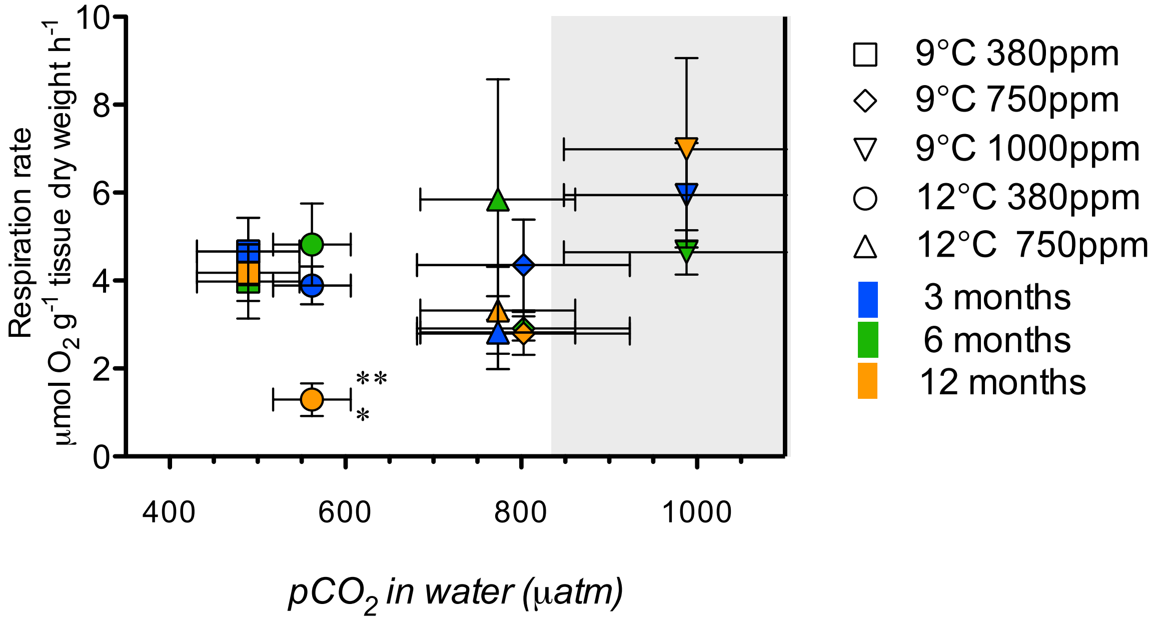

Supplementary Information Figure 3: *Respiration rates (µmol O_2 g^{-1} dry tissue mass h^{-1} ± SE, n = 8) at 3, 6 and 12 month time points from all experimental treatments. * and ** denote significant differences at that time point between and within treatments respectively. The shaded area represents water conditions where $\Omega_{Aragonite}$ <1.*
